# Testing the Youth Physical Activity Promotion Model during the COVID-19 Pandemic, with Partial Least Squares Second-Order Latent Constructs

**DOI:** 10.3390/ijerph18126398

**Published:** 2021-06-13

**Authors:** Elena Druică, Rodica Ianole-Călin, Monica Sakizlian, Daniela Aducovschi, Remus Dumitrescu, Robert Sakizlian

**Affiliations:** 1 Centre for Applied Behavioral Economics, University of Bucharest, 030018 Bucharest, Romania; elena.druica@faa.unibuc.ro (E.D.); rodica.ianole@faa.unibuc.ro (R.I.-C.); 2 Department of Physical Education, University of Bucharest, 030018 Bucharest, Romania; daniela.aducovschi@unibuc.ro (D.A.); remus.dumitrescu@unibuc.ro (R.D.); robert.sakizlian@unibuc.ro (R.S.)

**Keywords:** Youth Physical Activity Promotion framework, students, Romania, COVID-19, health policy-making

## Abstract

We tested the Youth Physical Activity Promotion (YPAP) framework on Romanian students in order to identify actionable determinants to support participation in physical activity. Our sample consisted of 665 responses to an online survey, with participants aged 18–23 (mean = 19 years); 70% were women. We used the partial least squares algorithm to estimate the relationships between students’ behavior and possible predictors during the COVID-19 pandemic. Our results indicate that all the theoretical dimensions of YPAP (predisposing, enabling and reinforcing) have a positive and significant impact on physical activity, with two mediating mechanisms expressed as predisposing factors: able and worth. Unlike previous research, we used second-order latent constructs, unveiling a particular structure for the enabling dimension that only includes sport competence, fitness and skills, but not the environmental factors.

## 1. Introduction

The promotion of physical activity (PA henceforth) is a demanding global public health challenge. Despite the growing awareness on the issue and the dedicated programs from the last decade [1,2], recent data show weak or no improvement at all in PA levels, but a worrying increase in sedentary behaviors in adults [3,4]. Both trends are associated with numerous negative health consequences, such as aggravating influences on non-communicable diseases (e.g., cardiovascular, cancers, diabetes, etc.) [5,6] and mental health problems [7,8]. Romania is illustrative for investigating PA: over one-half of its population declares a lack of engagement in PA [9], and half of all deaths are traceable to behavioral risk factors, with 4% attributable to low PA [10]. The restrictions imposed during the COVID-19 pandemic severely limited outside physical movement and social gatherings (e.g., the closure of theatres, restaurants and fitness clubs for some periods). Remote work, online learning and staying indoors became the default option supported by the official public health recommendations. All these measures had a further toll on physical activity, generating an additional layer of adverse outcomes on lifestyle and wellbeing [11,12], for most age groups. Furthermore, there are significant concerns that this period may reinforce, in an unprecedented manner, unhealthy behaviors in children and adolescents: unbalanced diets, higher anxiety levels, disrupted sleep schedules and passive screen time [13,14]. These concerns extend to university students [15], already considered an at-risk group for sedentary behavior prior the pandemic [16]. Recent work confirms that their PA levels have further decreased during the lockdown in Italy, Spain, the United Kingdom and Switzerland [17,18,19,20]. In Romania, PA classes were held online, with the inherent limitation of professors not being able to check if students actually exercised. Self-control failure literature [21] strongly suggests a high probability that students choose the comfortable option in the present, namely, not doing the exercises. In this completely unusual context, PA promotion strategies should be redesigned to accurately reflect the new relationships of individuals, both with themselves and with the physical and sociocultural environments.

To serve this practical purpose, our paper provides an exploration of the relationships between PA and its determinants, as specified by the Youth Physical Activity Promotion (YPAP) model. We aimed to identify actionable variables and potential structural mechanisms that support PA, directly and indirectly. The YPAP model [22] is a comprehensive theoretical framework [23] that looks at the interplay of predisposing, enabling and reinforcing factors upon PA [24,25]. These dimensions cover a blend of psychological variables simultaneously. One of YPAP’s major strengths is that it allows the inclusion of related constructs from different theoretical approaches [24], making it versatile and easy to adapt to different conditions. The core format includes first predisposing factors answering two main questions: “Am I able to do PA?” (simplified as the Able dimension); and “It is worth doing PA?”(simplified as the Worth dimension). The items appraise the degree of control that one perceives to have on their behavior in relation to PA, with respect to the motivations and beliefs about the target behavior of PA. We used the operationalization of [25] and included Self-Efficacy and Perceived Competence for the Able dimension, as well as Attitudes and Enjoyment for the Worth dimension. The second category, enabling factors, reflects upon biological (sport competence, fitness and skills) and environmental attributes. Finally, the reinforcing component comprises social influences that reward or encourage a desired behavior, expressed as peer support and role models (a refinement for young adults, by comparison to the parental support considered central for children [26]). All these constructs have been assessed, independently or partially connected, as influences for developing PA habits [27]. Traditionally, the model has been tested with structural equation modeling, explaining a large degree of variation in PA, of approximately 40–45% [22,28]. YPAP has largely been applied to children and adolescent populations (elementary, middle and high school), with a more recent interest for young adults and university students [29]. This scope requires further empirical proof for the performance of the model in its entirety. 

Our contribution is multifold. First, we consolidated the existing support for the YPAP model on university students and we expand the previous literature through an up-to-date methodological approach: first- and second-order models developed with the partial least squares method. Secondly, both population (students at the University of Bucharest, Romania) and period (during the COVID-19 pandemic) introduced new contexts to test the YPAP model. On the one hand, there have been no studies on PA in Romania, where, as much as in any other country, students are a critical category for long-term impact interventions. This is because studentship is generally a transitional stage towards the formation of new habits and routines in multiple life areas [30,31]. On the other hand, the YPAP has been tested in well-established contexts, with no behavioral constraints. Currently, people’s behaviors are at the opposite end, with the COVID-19 pandemic affecting not only the environmental conditions supporting physical activities, but also its main determinants. For instance, the perception on academic self-efficacy was influenced by the increased levels of anxiety of college students [32], suggesting a similar association for the enabling and predisposing factors impacting PA. Moreover, social distancing has decreased the strength of the reinforcing factors, such as peers and role models. Thus, this makes the pandemic a unique high-risk health context to test the applicability of the overall YPAP framework and to prepare mitigation strategies accordingly.

## 2. Materials and Methods

### 2.1. Data Collection

We collected data via an online questionnaire. Four professors of the Department of Sports and Physical Education of the University of Bucharest mainly disseminated the survey, and it was also posted on student groups. We targeted only students at the University of Bucharest. The minimum sample for a significance level of 0.05 and a power level of 0.990 was 407 if calculated using the inverse square root method and 385 if calculated using the gamma-exponential method [33]. 

The Ethical Committee of the University of Bucharest approved the research (decision no 22/01.05.2021). The respondents provided implicit consent participate in the study: at the beginning of the questionnaire, we mentioned that participation was voluntary and anonymous, and that by completing the questionnaire, they agreed to be part of this research. 

### 2.2. Measurement

Our dependent variable was PA participation, measured through the Leisure Time Exercise Questionnaire [34]. The items ask about the frequency of participation in different types of PA: on a 7-day interval, how many times do you engage in (1) PA that increases the rhythm of your breath and your pulse (unspecified duration); (2) mild PA, such as easy walking, for at least 15 min; (3) moderate PA, such as fast walking, for at least 15 min; and (4) intense PA, such as jogging, for at least 15 min. The first item was measured on an ordinal scale 1–3, where 1 means “never”, 2 means “sometimes”, and 3 means “very often”. We recorded the frequencies reported by the respondents to questions 2, 3 and 4, as numbers between 0 (never) and 7 (every day). In addition, and following previous studies that recommend this combination of items [25,35], we asked participants whether they regularly engaged in at least 150 min of moderate-intensity PA per week. The answers were recorded as “Yes” (coded as 1) or “No” (coded as 0). 

Our independent variables were provided by the YPAP framework as predisposing, enabling and reinforcing factors, and were measured using a previously validated questionnaire [25]. The YPAP framework captures a mixture of individual, social and environmental determinants of PA. Table 1 shows the items and the latent constructs along with their acronyms. We measured all responses on a 1–7 Likert scale, with 1 = complete disagreement, and 7 = complete agreement. We also included demographic characteristics—gender, education, age, weight and height, because they have been proven relevant in predicting PA among children, adolescents and youths [22]. Based on these objective characteristics, we also derived the body mass index (BMI), considering its consistent significance in relation to PA and different health risks (diabetes [36], hypertension [37], obesity [38]. BMI is particularly investigated in adolescent samples [39], although its importance has also been acknowledged for university students, because they are still in a formative stage that heavily influences their future adult life patterns [40].

### 2.3. Method

Given the lack of normality of our data, we employed a partial least squares structural equation modeling approach (PLS-SEM) [41]. PLS-SEM not only handles non-normally distributed data, but it also has the option of two different types of measurement for latent constructs: reflective and formative. Furthermore, PLS-SEM provides information about the amount of variance of the result explained by the predictors, and it also identifies the most suitable variables that can be addressed in practical interventions. Consequently, PLS-SEM better serves our purposes, compared with cov-based SEM, a method that is confirmatory in nature and it relies heavily on multivariate normality. 

PLS-SEM is an iterative algorithm that consists of two parts: a measurement model, also known as the outer model, that results in scores of the latent constructs; and a structural model, also known as the inner model, that assesses the relationships among variables. We conducted our analyses in WarpPLS version 7.0. Unlike other available software able to conduct similar estimations, WarpPLS captures potential non-linear relationships within the model by identifying the best curve that fits the data. 

Due to each predictor of the YPAP model being a multidimensional construct, we built two measurement models. First, we derived the scores of the latent constructs, as presented in Table 1, using a PLS regression algorithm, and we checked for item loadings. We found that the following items had loadings lower than 0.7 and could not be kept in the analysis: (1) “On an interval of 7 days, how many times do you engage in physical activity of mild intensity, such as yoga, easy walk, for more than 15 min?”—item of the PA construct; (2) “In my neighborhood there are bicycle/pedestrian lanes that I can easily access.” and “The sidewalks in my neighborhood are well maintained” as part of the environmental factors construct; and (3) “Many people around me are physically active” as part of the role model construct. After removing these items, we re-estimated the measurement model and extracted the scores of the latent constructs, aligned this time with the theoretical recommendations. Table 1 reports the amount of variance explained, as well as the internal consistency of each YPAP construct. All the measurements had good internal consistency, higher than the accepted threshold of 0.7 [42], and the amount of variance explained by each construct was above 0.5, as has previously been recommended [42,43]. All loadings were higher than 0.7, and they were higher than the corresponding cross-loadings. In addition, because none of the off-diagonal values were higher than 0.8, we retained all the variables in the model [44] and we confirmed that the first-order measurement model fitted the theoretical recommendations.

In the second stage, we created new latent constructs in line with the indications presented in Table 1. Able was created using the latent constructs of perceived competence and self-efficacy; Worth was composed of attitudes and enjoyment; Enabling comprised sports competence, fitness and skills, as well as environmental factors; and Reinforcing included peer-support and role model. After fitting the second-order model, we identified that environmental factors had a very low loading (0.540); thus, we removed this dimension from the enabling dimension. 

The last stage was to fit the structural model using the Warp3 inner algorithm, which allows for the identification of potential non-linear relationships among variables, and the resampling method Stable 3, to ensure the statistical inference [45,46]. 

## 3. Results

Our data comprised 665 respondents, aged 18–23 (median age 19.6, mean age 19, sd = 1). The body mass index ranged between 13.7 and 35.4 (median 21.5, mean 23.3, sd = 3.5), with 18.6% of the respondents underweight, 66.8% with normal weight, 11.7% overweight, and the rest of 2.86% with obesity. Out of the total sample, 70% were women and 30% were men. The description of the sample is reported in Table 2.

Table 3 presents the consistency of the second-order measurement model, showing that the convergent validity held. One second-order factor (Reinforcing) had a Cronbach’s alpha value below the recommended threshold of 0.7, but considering that this was an exploratory study and the first study in Romania, this value could be accepted [42,43]. Moreover, the composite reliability index for this construct was 0.841, above the threshold, and many authors consider this measure of internal consistency as more reliable than the Cronbach’s value. The rest of the values were above the recommended thresholds for Cronbach’s alpha, for the composite reliability indices and for the average variance extracted. In addition, Table A1 in Appendix A shows that the diagonal loadings within each construct were higher than 0.7, and that the diagonal blocks had higher values than the non-diagonal blocks of loadings. Thus, it was confirmed that the discriminant validity held. 

In Table 4, we report the correlations among latent variables, with square roots of AVEs on the main diagonal. None of the off-diagonal values are higher than the diagonal values, which further supports divergent validity. 

**Figure 1:**
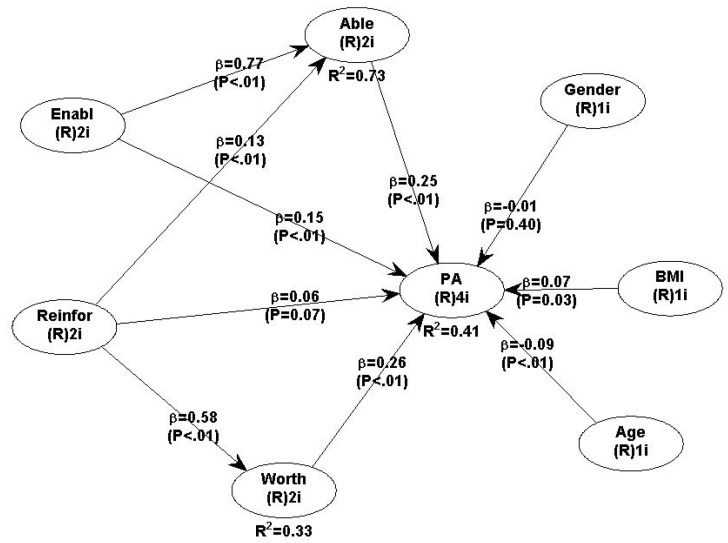
The structural model that explains PA, as proposed by [25].

Table 5 presents the results of the model that explains PA as a function of the four latent determinants (Able, Worth, Reinforcing and Enabling) and two structural mechanisms (Able and Worth) as mediators. The same information is presented in Figure 1 in a more intuitive manner. 

### 3.1. Demographic Variables

According to Table 5, age was negatively correlated with PA (β = −0.090, *p* = 0.010); there were no gender differences in predicting the amount of time the respondents spent exercising (β = −0.010, *p* = 0.398); BMI was positively correlated with PA (β = 0.075, *p* = 0.026). 

### 3.2. The YPAP Predictors

Using the YPAP framework, we had information for four predictors, two predisposing factors, Able and Worth, developed as the mechanisms that explained the relationship between the Enabling and the Reinforcing factors, and PA. Our results indicate that all four factors were important in the architecture of the YPAP and that they were positively related with PA. 

#### Total Effects

The respondents’ perceived competence in PA matters, and their sense of self-efficacy was captured by the independent variable Able that predicted PA (β = 0.249, *p* < 0.001). The participants’ attitudes towards PA, and the enjoyment they experienced while engaging in it, were captured by the variable Worth, which also predicted PA (β = 0.259, *p* < 0.001). Equally significant predictors were the Reinforcing factors (β = 0.241, *p* < 0.001), and the Enabling factors (β = 0.343, *p* < 0.001). 

### 3.3. Total Effect Decomposition

After controlling for two mediators, Able and Worth, we found that the direct effect between Reinforcing and PA became statistically insignificant (β = 0.058, *p* = 0.065), whereas the indirect effect via the mediators was statistically significant (β = 0.183, *p* < 0.001). This shows that Able and Worth completely mediated the relationship between Reinforcing and PA. In a similar vein, we decided that Able mediates the relationship between Enabling and PA, due to the statistically significant indirect effect ((β = 0.191, *p* < 0.001), although this is a partial mediator: after controlling for Able, the direct effect of the Enabling factors and PA remained statistically significant (β = 0.151, *p* < 0.001). 

As Table 5 presents, the model had a very good explanatory power. Enabling and Reinforcing factors explained 73% of the variance of the predisposing factor related to the respondent-perceived competence and self-efficacy in enabling in physical education, Able. The Reinforcing factor alone explained 34% of the variations in Worth. Overall, the YPAP variables explained 41.3% of the variation in the amount of time the respondents engaged in PA. 

Table 6 presents the effect sizes of each YPAP predictor using the equivalent of Cohen’s f2 within the context of PLS-SEM, under the assumption of linear relationships. Effect sizes larger than 0.02 are considered large enough to justify recommendations for practical intervention [47]. Below this threshold, the effect sizes are not practically relevant, even though the relationship they describe may be statistically significant [48]. Table 6 shows that Able and Worth were not only statistically significant in predicting PA, but also had effect sizes of sufficient magnitude to support practical interventions (0.145 for Able, and 0.142 for Worth). The same applied to Enabling in relation to Able (effect size = 0.651, the highest effect size in Table 6), and to Reinforcing in relation to Worth (effect size = 0.334). 

**Figure 2:**
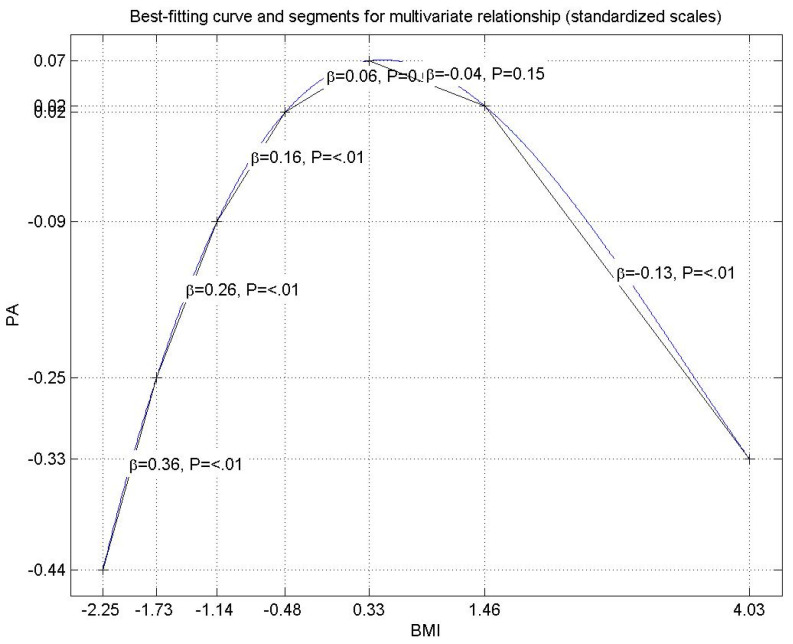
The non-linear relationship between the amount of time people engage in physical activities (PA) and body mass index (BMI).

There is one result presented in Table 5 that deserves more in-depth consideration. This result, derived under the assumption that the relationships within the model were linear, shows that as BMI increases, the amount of time spent in engaging in PA increases too. Figure 2 shows, however, that the relationship between these two variables is best described by a non-linear curve. There are four regions to be discussed: the first region refers to the respondents with standardized BMI values lower than −0.48 where the relationship between BMI and PA is indeed direct and statistically significant (β = 0.36; β = 0.26; β = 0.16, *p* < 0.001 in all three cases), although the beta coefficients decrease as BMI increases. The second region pertains to people with standardized BMI values between −0.48 and 0.33, where the estimated beta is positive, β = 0.06, although not statistically significant. The third region regards standardized BMI values between 0.33 and 1.46; this region shows a negative relationship between BMI and PA (β = −0.04), although not statistically significant. The last region includes standardized BMI values higher than 1.46, where the relationship is negative and statistically significant (β = −0.13, *p* < 0.001). These results show that the relationship between BMI and the engagement in PA is more complex than it may appear, and that practical interventions should be tailored to carefully address the groups at risk.

## 4. Discussion and Conclusions

Our paper examined what predicts the amount of time students engage in PA, using the YPAP framework. Our results confirm previous findings showing that PA is strongly determined by predisposing, enabling and reinforcing factors [25], with a similar explanatory power (41.3% overall, in the range of 40–45% suggested as an average in the literature [28]). Thus, we have proved the external validity of the YPAP model in Romania and that its robust predictions also hold for the period of the COVID-19 pandemic.

The use of structural models offers further clarification on the internal mechanisms leading to variations of the PA levels: Able and Worth (the dimensions of Predisposing factors) play the mediators of the relationships between Enabling and Reinforcing factors and PA. This means that interventions targeting the constructs of Reinforcing and Enabling will not directly affect the amount of time people exercise, but they will develop their perception of competence and self-efficacy, captured by the mediator Able, and their sense of enjoyment, while exercising is captured by the mediator Worth. In turn, these developments will improve the amount of time students engage in PA. The two mediators are consistent with the literature that highlights the major role of self-efficacy in PA youth interventions, followed by attitudes and enjoyment [49,50]. 

There are, however, some inherent particularities in the structure of the latent variables, unveiled by analysis of the first-order model. For instance, the item referring to PA of mild intensity (such as yoga, easy walk) was dropped from the structure of the PA variable (loading lower than 0.7), suggesting a low popularity of these activities or even a lack of association with PA. The result may be explained through the relatively poor level of knowledge about the health benefits of both mild and more intensive types of PA, observed in other studies for Romanian university students, levels similar to those registered in Thailand and South Africa [51]. There is reason to also consider this behavior as the result of a cultural norm that originates from the marginal treatment received by PA as a school topic (such as a reduced number of dedicated hours, inappropriate placement in daily/weekly schedule [52]), with a clear emphasis on teachers and parents favoring the development of academic skills in comparison to motor skills/healthy habits. This deserves attention for the purpose of creating appropriate country-level interventions, considering (i) the importance of gradual approaches in building habits, from mild to more intense programs; and (ii) the evidence showing other type of core benefits of these specific physical activities, such as yoga’s positive influences on students’ capacity to develop cognitive and psychological traits associated with entrepreneurship [53,54]. 

A similar discussion appears in relation to the items excluded (also due to low loadings) from the environmental factors, within the enabling dimension. These items refer to the existence of bicycle/pedestrian lanes in the neighborhoods with respect to the quality of sidewalks. Again, this is less surprising considering the infrastructure of the capital city of Bucharest, which, similar to other post-communist cities in Central and Eastern Europe, was more focused on industrial development than on the quality of life in residential areas [55,56], focusing on automobiles and not pedestrian areas [57]. Thus, raising awareness on the objective improvements needed in the city’s infrastructure can be performed more convincingly if such improvements are presented linked to the positive behavior changes in PA.

Finally, the role model construct was validated without the item stating that “Many people around me are physically active”, suggesting that the social environment does not reflect the existence of a normative influence on PA. The finding is relevant because it shows that, at this point, there is no fertile ground for simply replicating social norm nudging interventions to increase PA, although they were successful in other contexts [58]. The findings of our study rather point out the need of understanding existing norms related to PA, namely, what beliefs and barriers have to be addressed for potentially developing new norms, more favorable to PA. This is especially relevant for young people that often emphasize, as revealed by systematic reviews, the social side of PA [59].

Overall, it is difficult to assess with precision to what degree the differences obtained in our study are due to the socio-cultural norms and urban infrastructure in Romania, or to the significant changes induced by the COVID-19 pandemic (less time spent outside, thus less time spent walking and fewer opportunities to observe the environment and to see if the people around engage in PA). 

The results also illustrate what may constitute the base of efficient interventions, with all the predictors having significant effect sizes and the enabling dimension being the most salient. This echoes previous findings that have emphasized the major role of fitness and fatness as enabling factors for PA [60], with a marked difference on what concerns environmental factors. In the second-order models, this category did not load highly enough in the enabling factors, therefore it had to be removed. This finding adds to the body of research still trying to pinpoint exactly the role of these factors for PA [61]. The key question is what type of environmental attribute is actually relevant for PA. As mentioned previously, the factor analysis suggested the elimination of two aspects considered crucial in other contexts: sidewalks and bicycles lanes. Thus, it may not be surprising that the remaining aesthetic reasons and green spaces were not proven as strong enough predictors. This is valuable for the local administrations of Bucharest’s districts because it points towards a gap in people’s perception of some general good measures (such as greening the city or focusing on pedestrians): they do not see the relevance of the measures for them personally, such as supporting their engagement in PA, and therefore improving their health and quality of life. An information campaign making this important association clearer may lead to more public support for the initiatives and to a better use of the spaces.

Reinforcing factors ranked in second position for interventions. It is unsurprising that aspects such as peer support and role models enhance the perceived worth of PA for students, as with adolescents and children [62]. The challenge is to build up a sense of social belonging though PA, complementary to what may be the default from implicit sedentary activities (e.g., attending class, studying, using the computer, etc.). For instance, the use of peer mentors in promoting PA has been proven as an effective strategy for increasing self-efficacy, the perception of competence, and self-determination [63]. Similarly, research on PA apps has illustrated competition systems with friends as a preferred feature, with transparent rewards and rankings [64]. Such strategies should serve as inspiration for official recommendations tailored at a national level and for programs initiated and/or endorsed by the education and health authorities.

In a top-down approach, the findings also open a unique opportunity for the university to take a more pro-active role in shaping the micro socio-ecological environment of the students. This may happen not only through facilities but also through a more oriented strategy towards the promotion of PA and health behaviors in general, under the aim of creating healthy universities [65]. Such an approach mirrors the strong evidence obtained for the effectiveness of school-based interventions in adolescents [66], with the difference that at the university level, its importance is acknowledged indirectly in the indicators tracked by different international rankings (e.g., health and wellbeing within the Sustainable Development Goals) [67].

On the control variables side, we identified a non-linear relationship showing that the connection between BMI and PA is more complex that a linear relationship. This provides a novel and convincing explanation for what was considered a rather counterintuitive result in other studies: a higher BMI was associated with more PA [25]. It also suggests a potential criterion for prioritizing interventions based on specific value intervals of BMI.

Our contribution is not without limitations. We used a convenience sample, representative only for students from the University of Bucharest, the second largest university in Romania. Thus, although there is ground for inferences on students from other large university centers, these should be drawn carefully, considering other potential confounders. In a similar vein, our sample was biased towards women (70% of the sample) and first-year students (average age 19). A theoretical extension should consider that YPAP does not explore the role of technology in PA promotion. Taking into account the emergence of gamification and smartphone applications focused on behavioral changes in general [68] and on PA in particular [69], this is probably a dimension that should be included and tested under this unified frame. Last, but not least, the questionnaire used to assess the YPAP dimensions in Romania has not previously been validated, which creates ground for future research in this area. 

## Figures and Tables

**Table 1:** Measurement items by YPAP construct.

Dimension	Items	Latent Construct	Variance Explained	Cronbach’s Alpha
Physical activity	On a 7-day interval, how many times do you engage in physical activity that increases the rhythm of your breath and your pulse (unspecified duration)?	Physical activity, PA	63.3%	0.806
	On a 7-day interval, how many times do you engage in moderate physical activity, such as fast walking, for at least 15 minutes?			
	On a 7-day interval, how many times do you engage in intense physical activity, such as jogging, for at least 15 minutes?			
	Do you regularly engage in at least 150 min of moderate intensity physical activity per week?			
Predisposing—Able	I think I am pretty good at physical activity	Perceived competence, PC	79.5%	0.893
	I am happy with my performance when I engage in physical activity.			
	After I engage in physical activity for some time, I feel I am pretty good at it.			
	I am pretty good at physical activity			
	How confident are you that you can engage in physical activity when you are tired?	Self-efficacy, SE	75.9%	0.893
	How confident are you that you can engage in physical activity when the weather is bad?			
	How confident are you that you can engage in physical activity when your program is very busy?			
	How confident are you that you can engage in physical activity when you have so many things to do?			
Predisposing—Worth	I find engaging regularly in physical activity boring (reversed).	Attitudes	85%	0.823
	When I must engage in physical activity, I feel I would do anything but this (reversed).			
	I like to engage in physical activity	Enjoyment	85.6%	0.958
	I find the physical activity pleasant.			
	I like very much to engage in physical activity; I feel completely absorbed in it.			
	When I engage in physical activity, I feel happy.			
	Engaging in physical activity is pleasant.			
Enabling	I am good at any type of sports or physical activity.	Sports competence, SC	81.5%	0.772
	I know how to organize my own physical activity program.			
	I am very confident regarding my fitness.	Fitness and skills, FS	92.9%	0.924
	I am very confident that I can keep myself in a good physical shape.			
	There is a lot of green in my neighborhood.	Environmental factors, EF	68.5%	0.769
	There are many beautiful buildings and places in the vicinity of my home.			
	There are many interesting things in my neighborhood, to be discovered when I walk.			
Reinforcing	My friends encourage me to engage in sport and physical activities.	Peer-support	65.7%	0.826
	My friends engage in sport and physical activities together with me			
	I encourage my friends to engage in sport and physical activities.			
	My friends tell me that I am doing well at sport and physical activities.			
	When I see people engaged in physical activity, I feel motivated to engage too.	Role-model	76.4%	0.690
	I admire people who are physically active.			

**Table 2:** Descriptive statistics of the sample.

Categorical Descriptors		Frequency			
Gender					
Female		70%			
Male		30%			
Body Mass Index					
Underweight		18.6%			
Normal weight		66.8%			
Overweight		11.7%			
Obesity		2.9%			
Numerical descriptors	Min	Mean	Median	Max	SD
Age	18	19	19.6	23	1
Body Mass Index	13.7	21.5	23.3	35.4	3.5

**Table 3:** The consistency of the measurement of the second-order model.

Second-Order Latent Construct	Predisposition—Able	Predisposition—Worth	Reinforcing	Enabling
Cronbach’s alpha (>0.7)	0.837	0.706	0.621	0.878
Composite reliability index (>0.7)	0.925	0.872	0.841	0.942
Variance extracted (AVE > 0.5)	86.0%	77.3%	72.5%	89.1%

**Table 4:** Correlations among latent variables, with square roots of AVEs on the main diagonal.

	PA	Able	Worth	Reinforcing	Enabling
PA	**0.795**	0.580	0.536	0.448	0.555
Able	0.580	**0.927**	0.625	0.592	0.849
Worth	0.536	0.625	**0.879**	0.577	0.596
Reinforcing	0.448	0.592	0.577	**0.852**	0.594
Enabling	0.555	0.849	0.596	0.594	**0.944**

**Table 5:** Estimated coefficients of the model with two mediators, explaining PA under the assumption of linear relationships.

Model	Direct Effects			Indirect Effect Via Mediators	Total Effect
	Predisposing—Able	Predisposing—Worth	Physical activity (PA)	-	Physical Activity (PA)
Able	-	-	0.249 *** (*p* < 0.001)	-	0.249 *** (*p* < 0.001)
Worth	-	-	0.259 *** (*p* < 0.001)	-	0.259 *** (*p* < 0.001)
Reinforcing	0.133 (*p* < 0.001)	0.578 (*p* < 0.001)	0.058 (0.065)	0.183 (*p* < 0.001)	0.241 *** (*p* < 0.001)
Enabling	0.768 (*p* < 0.001)	-	0.151 (*p* < 0.001)	0.191 (*p* < 0.001)	0.343 *** (*p* < 0.001)
Age	-	-	−0.090 * (0.010)	-	−0.090 * (0.010)
BMI	-	-	0.075 * (0.026)	-	0.075 * (0.026)
Gender Female Male	-	-	Reference −0.010 (0.398)	-	Reference −0.010 (0.398)
R^2^/Adjusted R^2^	73.1%/73.1%	33.4%/33.3%	41.3%/40.7%	-	-
Tenehaus GoF (small >= 0.1, medium >= 0.25, large >= 0.36)				0.651	

*p*-values in parentheses; -*p* < 0.10; *—*p* < 0.05; ***—*p* < 0.001.

**Table 6:** Effect sizes of the direct effects—actionable predictors of physical activity.

Model	Able	Worth	Physical Activity (PA)
Able	-		**0.145**
Worth	-		**0.142**
Reinforcing	0.080	**0.334**	0.027
Enabling	**0.651**	**-**	0.084
Age	-	-	0.004
BMI	-	-	0.010

## Data Availability

Data is available on request.

## Appendix A

**Table A1:** Combined loadings and cross-loadings of the variables involved in the second-order measurement model.

	PA	Able	Worth	Reinforcement	Enabling
PA1	**0.795**	0.240	0.030	−0.045	−0.061
PA2	**0.833**	0.111	−0.013	−0.062	−0.046
PA3	**0.780**	−0.159	−0.069	0.047	0.017
PA_tota	**0.772**	−0.206	0.053	0.066	0.095
lv_PC	−0.012	**0.927**	0.080	0.019	0.119
lv_SE	0.012	**0.927**	−0.080	−0.019	−0.119
lv_BORE	0.051	−0.393	**0.879**	−0.162	0.025
lv_ENJO	−0.051	0.393	**0.879**	0.162	−0.025
lv_Role	0.002	0.095	0.232	**0.852**	−0.467
lv_PS	−0.002	−0.095	−0.232	**0.852**	0.467
lv_SC	−0.038	−0.034	−0.025	0.033	**0.944**
lv_FS	0.038	0.034	0.025	−0.033	**0.944**
